# Transition from child and adolescent MHS to adult MHS: what happens to young people with personality disorder?

**DOI:** 10.1192/bjo.2021.154

**Published:** 2021-06-18

**Authors:** Martynas Malkov, Tennyson Lee, Hanspeter Dorner, Alana Ahmet, Alzbeta Karlikova, Kamaldeep Bhui, Andrew Chanen

**Affiliations:** 1 Tavistock and Portman NHS Foundation Trust; 2 East London NHS Trust; 3 Professor of Cultural Psychiatry & Epidemiology, Centre Lead for Psychiatry, Wolfson Institute of Preventive Medicine, Hon. Consultant Psychiatrist, East London Foundation Trust, Barts & The London School of Medicine & Dentistry, Queen Mary University of; 4 Orygen

## Abstract

**Aims:**

*Hypothesis:* Personality Disorder (PD) adolescents, compared to non-PD case, have a worse experience at transition.

*Aims:* To describe the outcomes of referrals of adolescents for transition to adult services and compare PD and non-PD populations to identify potential improvements to allow for better transition experience of the PD patients.

**Background:**

Borderline PD is prevalent in adolescents - although there is a reluctance to make the diagnosis. When patients reach graduation from CAMHS, many fall through the ‘gap’ in services during the transition. Consequently, adding the paucity in research about the transition experience of PD patients, it is important to evaluate what happens to these patients during the transition process to help better understand their experience, and how it can be improved.

**Method:**

Patient's clinical records from Tower Hamlet CAMHS, East London NHS Foundation Trust, were reviewed retrospectively from July 2018 to November 2019, assessing whether optimal transition standards were met. A total of 41 cases that transitioned from CAMHS to AMHS were identified. Transition standards compared were: information sharing – case and risk, parallel care, transition planning and continuity. PD diagnosis was identified based on the recording of this diagnosis or meeting DSM5 criteria from the notes. PD and non-PD transition experience was compared.

**Result:**

36 were given a diagnosis by the CAMHS clinician at transition and 5 had no diagnosis assigned. No cases had a PD diagnosis made by the CAMHS clinician, however 1 case mentioned ‘PD traits’, 1 mentioned ‘EUPD’ as a possible differential and 2 cases were labelled as ‘emotional dysregulation’. The research team found 17 cases that met DSM5 criteria for PD diagnosis.

Comparing transition experience of PD vs non-PD patients, the PD patients had a less optimal transition process. Statistical analysis using Chi Square Tests, showed significantly less optimal transition planning (X2 = 5.103, p < 0.05) and continuity (Fisher's exact test p = 0.049). Cohens W indicated a medium effect for transition planning and continuity.

**Conclusion:**

Adolescents with a diagnosis of PD transition less well to Adult MHS than those without the PD diagnosis. Implications of our findings point to 1) the importance of considering a diagnosis of PD 2) if the diagnosis of PD is made, to anticipate greater difficulties in transition 3) the need to identify specific reasons for transition difficulties related to patient, clinician and system factors and their interrelation.

